# Gas sensing properties of individual SnO_2_ nanowires and SnO_2_ sol–gel nanocomposites

**DOI:** 10.3762/bjnano.10.136

**Published:** 2019-07-08

**Authors:** Alexey V Shaposhnik, Dmitry A Shaposhnik, Sergey Yu Turishchev, Olga A Chuvenkova, Stanislav V Ryabtsev, Alexey A Vasiliev, Xavier Vilanova, Francisco Hernandez-Ramirez, Joan R Morante

**Affiliations:** 1 Voronezh State Agrarian University, Michurina, 1, Voronezh, 394087, Russia; 2 Voronezh State University, Universitetskaya, 1, Voronezh, 394018, Russia; 3 NRC Kurchatov Institute, Kurchatov Sq. 1, 123182, Moscow, Russia; 4 Rovira i Virgili University, Av. Paisos Catalans 17-19, 43007, Tarragona, Spain; 5 World Sensing, S.L., Viriat 47, 10th floor, 08014 Barcelona, Spain,; 6 Catalonia Institute for Energy Research, Sant Adrià de Besòs, Catalonia, Barcelona, 08019, Spain

**Keywords:** gas sensors, gas transport method, nanowires, quasi-one-dimensional materials, sol–gel synthesis, tin dioxide, X-ray absorption near edge structure (XANES), X-ray photoelectron spectroscopy (XPS)

## Abstract

This work is an investigation of the properties of semiconductor materials based on metal oxides, their catalytic properties, and their application as gas sensors, which were shown to exhibit high sensitivity, stability, and selectivity to target gases. The aim of this work is the comparison of gas sensing properties of tin dioxide in the form of individual nanowires and nanopowders obtained by sol–gel synthesis. This comparison is necessary because the traditional synthesis procedures of small particle, metal oxide materials seem to be approaching their limit. Because of this, there is increasing interest in the fabrication of functional materials based on nanowires, i.e., quasi-one-dimensional objects. In this work, nanocrystalline tin dioxide samples with different morphology were synthesized. The gas-transport method was used for the fabrication of well-faceted wire-like crystals with diameters ranging between 15–100 nm. The sol–gel method allowed us to obtain fragile gels from powders with grain sizes of about 5 nm. By means of X-ray photoelectron spectroscopy (XPS) it was proven that the nanowires contain considerably smaller amounts of hydroxy groups compared to the nanopowders. This leads to a decrease in the parasitic sensitivity of the sensing materials to humidity. In addition, we demonstrated that the nanowires are characterized by a nearly single-crystalline structure, ensuring higher stability of the sensor response due to the unlikelihood of sample recrystallization. The results from the ammonia detection experiments showed that the ratio of the sensor response to the surface area exhibits similar values for both the individual nanowire and nanopowders-based sensor materials.

## Introduction

Semiconductor sensor functionality relies on heterogeneous catalytic chemical processes, which makes the surface-to-volume ratio of gas sensing materials an important parameter in determining their gas sensitivity. Traditionally, quasi-0-dimensional (i.e., spherical) nano-objects have been used in order to create highly porous materials. In gas sensors, agglomerates of nanoparticles with a high specific area and high surface-to-volume ratio, obtained by sintering, are traditionally used as sensing materials. By means of preparation methods such as magnetron sputtering, laser ablation, and pulverization, layer-by-layer nanoparticle deposition can be achieved with adhesion to the substrate and to previously formed material. Sol–gel processes comprise the synthesis of nanopowders, consisting of spherical nanoparticles, the preparation of pastes from these powders, and finally, deposition and annealing.

The development of the sol–gel synthesis of small particle semiconductor materials is no longer a mainstream process in sensor development, because researchers have reached the limits of this method. For this reason, the interest in the development of nanowire devices (i.e., quasi-1-dimensional objects) has increased. Their surface-to-volume ratio can be as high as that of nanopowders obtained from spherical nanoparticles.

The first nanowires were synthesized in the 1960s, but their widespread application only started in the beginning of the 21st century, when advancements in technology required the development of a wide range of nanomaterials and new methods for their treatment. The basic method for nanowire synthesis was developed and reported in detail in the classic work of R. Wagner and W. Ellis [1]. Recently, with the use of this method, SnO_2_, In_2_O_3_, WO_3_, ZnO and other metal oxide nanowires were obtained [1–5]. Liquid phase synthesis methods have also been widely implemented [6–9].

The use of metal oxide nanowires as sensing elements in gas sensors continues along two directions: The first direction involves the use of large quantities of nanowires or “nanosponge”. For example, nanowires can be grown on the surface of metallic electrodes deposited on a dielectric substrate, wherein random electrical contact between wires located on different electrodes are formed. The contact between each pair of nanowires is not stable, but due to the large number of contacts, completely stable electrical contact behavior is observed (on a statistical average). Sensors based on such systems show high sensitivity [10–20]. Hierarchical structures with SnO_2_ nanowires covered with additional nanoscale objects can be used for the improvement of electrical contacts [11,16–17]. A second direction in nanowire sensor development is the manufacturing of electrical contacts with individual nanowires [21–33]. These contacts can be made by means of photolithography, but more often, focused ion beam (FIB) technology is used for this purpose. This approach has several advantages: first, a reliable electrical contact between the nanowires and electrodes is provided; secondly, the possibility for the manufacture of devices with ultralow energy consumption opens up.

In the case of individual nanowires, two pairs of electrodes are deposited onto the nanowire. The outer pair is used for applying electrical heater current, while the inner pair is used for the measurement of the electrical potential drop. This 4-electrode scheme of electrical resistance measurement improves the quality of sensor response detection.

In spite of the large number of works dedicated to the use of nanowires as conductometric gas sensors, these have not been compared in detail with classical semiconductor sensors manufactured by means of the sol–gel method. Some results related to this comparison obtained by Dr. Dmitry Shaposhnik were used in his PhD thesis performed under the supervision of Professor X. Vilanova at the University Rovira I Virgili (Tarragona, Spain). The preliminary results concerning this publication were discussed at Eurosensors conferences [32–33]. The present work presents a comparative study of the material properties of SnO_2_ devices prepared by different methods and by using ammonia as a reference gas for the assessment of their sensing characteristics. The use of ammonia in recent works is due to the strong interest in this gas as a marker for stomach diseases and to our collaboration with a company producing suitable measurement instruments in Russia (St. Petersburg).

## Experimental

### Material synthesis and characterization

#### SnO_2_ nanowire synthesis

The gas transport method based on the vapor–liquid–solid (VLS) mechanism was used for the synthesis of SnO_2_ nanowires. Argon saturated with water vapor served as the transport medium. Water was used as a mild oxidant of metallic tin in the following reaction:

[1] $$\text{Sn}+2{\text{H}}_{\text{2}}\text{O}\to {\text{SnO}}_{\text{2}}+2{\text{H}}_{\text{2}}$$

The formation of SnO_2_ is a result of a cascade of oxidation processes. It is necessary to note that in addition to the completely oxidized form of Sn (SnO_2_), in gas flow there are products of the incomplete oxidation of tin, for example, SnO. On the substrate, this oxide decomposes following a disproportionation reaction as

[2] $$2\text{ SnO }\to \text{ Sn }+{\text{ SnO}}_{\text{2}}$$

Metallic tin forms on the surface nanodrops, dissolving tin dioxide and the products of the incomplete oxidation of tin (SnO, Sn_2_O_3_, Sn_3_O_4_) from the argon flow. This dissolution leads finally to the saturation of tin with tin dioxide that is stable at high temperature. After this saturation occurs, the thin dioxide nanowires begin to crystallize. The diameter of the growing wires is defined by the diameter of the initial tin nanodrops. Such a process, taking place without hetero-element clusters, is called a self-catalytic process. A nanodrop of liquid metal with dissolved tin oxide is located on tip of the growing nanowire. This location will be responsible for the nanowire propagation following the VLC mechanism. This self-catalytic mechanism was described in detail in [34].

**Figure 1:**
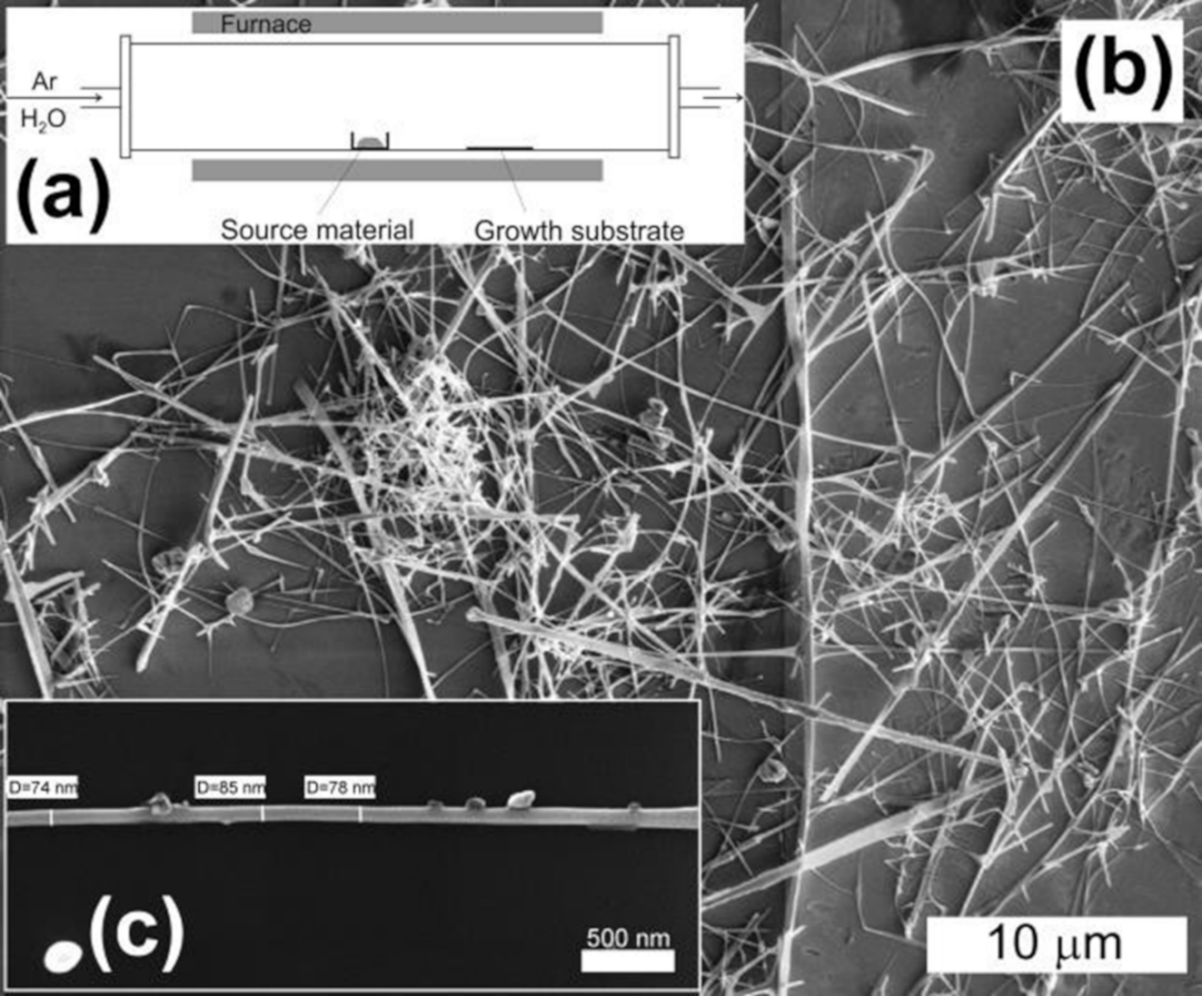
Scheme of nanowire synthesis (a), SEM image of SnO_2_ nanowires after synthesis (b), SEM image of a single SnO_2_ nanowire (c).

The temperature of the metal source in a tube furnace (Figure 1a) was fixed at 1100 °C. The nanowires grew on the surface of the quartz tube and on quartz substrates (Figure 1a,b). The diameter of the nanowires ranged between 15 and 150 nm (Figure 1c).

#### SnO_2_ nanopowder synthesis

The precipitation method, reported in [30], was used to synthesize nanodispersed tin dioxide. Tin(II) acetate was dissolved in glacial acetic acid. A surplus of hydrogen peroxide was added to the solution:

[3] $$\begin{array}{l} {\text{Sn(CH}}_{\text{3}}{\text{COO)}}_{\text{2}}+{\text{H}}_{\text{2}}{\text{O}}_{\text{2}}+2{\text{CH}}_{\text{3}}\text{COOH}\\ \;\to {\text{Sn(CH}}_{\text{3}}{\text{COO)}}_{\text{4}}+2{\text{H}}_{\text{2}}\text{O} \end{array}$$

where NH_3_·H_2_O was added dropwise to cause hydrolytic precipitation of tin oxide as follows:

[4] $$\begin{array}{l} {\text{Sn(CH}}_{\text{3}}{\text{COO)}}_{\text{4}}+4{\text{NH}}_{\text{3}}+3{\text{H}}_{\text{2}}\text{O}\\ \;\to {\text{H}}_{\text{2}}{\text{SnO}}_{\text{3}}↓+\;{\text{4CH}}_{\text{3}}{\text{COONH}}_{\text{4}} \end{array}$$

The obtained colloid was precipitated by centrifugation, dried and annealed. Tin dioxide nanopowder was formed as a result of tin acid calcination as follows:

[5] $${\text{H}}_{\text{2}}{\text{SnO}}_{\text{3}}\;\to {\text{SnO}}_{\text{2}}\;+{\text{H}}_{\text{2}}\text{O}$$

**Figure 2:**
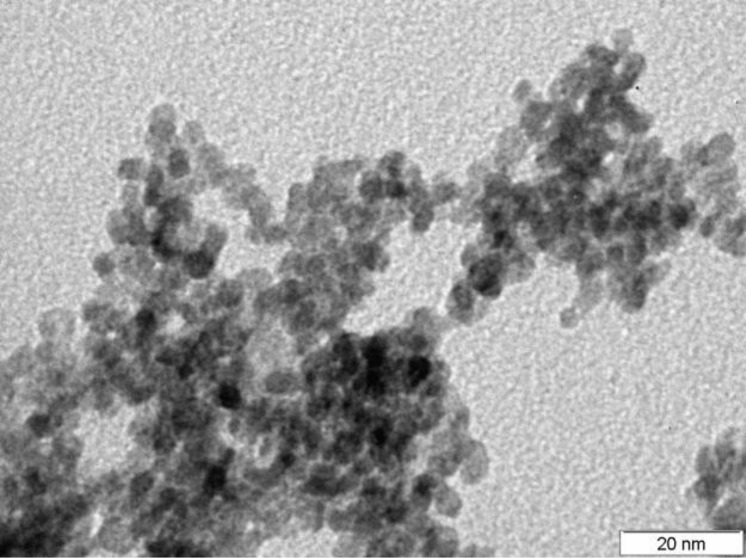
TEM image of blank tin dioxide nanopowder after annealing.

Figure 2 shows a TEM image of the obtained material. The particle diameter derived from this measurement was found to be 4–6 nm.

#### X-ray spectroscopy of the materials

In the present study, we used the non-destructive techniques, X-ray photoelectron spectroscopy (XPS) and X-ray absorption near edge structure (XANES), both employing the high-brilliance synchrotron radiation of the BESSY II storage ring at the Helmholtz Zentrum Berlin on the joint Russian–German beamline. The efficiency of XPS and XANES has previously been demonstrated in investigations of local atomic surroundings, specifically in nanomaterials, and particularly in the investigation of the structure of tin–oxygen systems [35–39]. These methods, in particular, demonstrated high sensitivity to the physical and chemical states of surfaces and interfaces. A sintered lump of tetragonal SnO_2_ (Alfa Aesar) was used as a reference.

The application of synchrotron radiation allowed the variation of X-ray quantum energies for the excitation of XANES spectra. These spectra represent local partial densities of free electronic states in the conduction band of the investigated materials [35–41]. The fine structure excited by ultrasoft X-ray (synchrotron) quanta close to a given atom’s core level absorption resonance has a very developed fine structure with all its features related to the density of electronic states. This at least allows for qualitative experimental information about the composition and the structure of the material surface layer to be obtained, sometimes accompanied by ab initio calculations [41–42]. According to the dipole selection rules Sn M_4,5_ (3d) XANES spectra represent transitions from core 3d states to free p- and f-states in the conduction band. Oxygen K (1s) spectra in turn represent transitions from the core 1s states of oxygen atoms to the free p-states in the conduction band.

XPS is a direct experimental technique allowing the detection of the charge state of the atoms. High energy resolution XPS spectra of core level chemical shifts can give information about chemical binding energy contributions in surface layers with complex phase compositions [43–47]. In the present study, we investigated core 1s states of oxygen atoms and core 3d states of tin atoms with high resolution provided by monochromatized synchrotron radiation. The spin–orbit splitting of tin 3d core levels gives us the possibility to detect Sn 3d_5/2_ states with high resolution.

The application of high intensity synchrotron radiation provides high-resolution signal detection from small samples with XANES and XPS methods using photon fluxes of 10^12^–10^13^ photons/sec at ring currents of 150–300 mA. The instrumental broadening was ≈0.1 eV. The pressure in the experimental and preparation chambers of the Russian–German lab end-station was ≈10^−10^ Torr.

The XPS analysis layer depth under 800 eV synchrotron radiation quanta (maximal flux) is estimated to be ≈1.5 nm [48].

In the case of XANES measurements, the analysis layer depth was ≈10 nm [49]. Calibration and normalization of the measured spectra was carried out using the signals from a pure gold film. Additionally, the positions of the core levels were controlled by the position of the C 1s level of the hydrocarbon contamination on the sample surfaces.

### Device manufacture

#### Manufacture of the device based on a single nanowire

**Figure 3:**
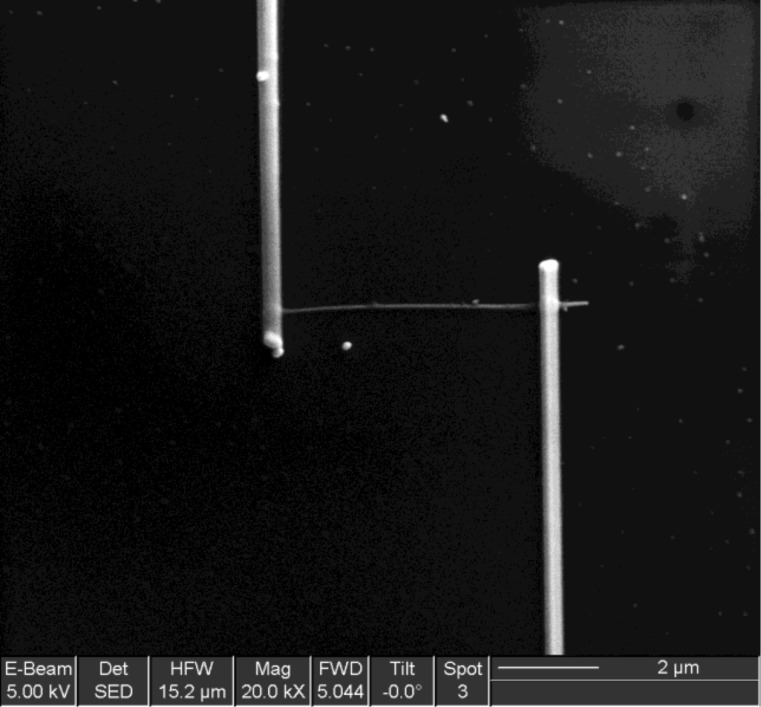
Nanowire with electrical contacts.

Individual nanowires were electrically contacted by direct focused-ion beam (FIB) platinum deposition, using an FEI dual beam Strata 235 instrument combined with a metal–organic injector to deposit platinum, following a process described elsewhere (Figure 3) [29]. The electrical measurements were performed using a Keithley 2400 source meter unit (SMU). For gas sensing experiments, the devices were placed in a Linkam chamber with an integrated heater; the gas flow (≥99.999% purity) was regulated by mass flow controllers.

#### Manufacture of the device based on sol–gel material

Tin dioxide nanopowder was mixed with ethylene glycol to obtain a paste which was deposited on a thin alumina substrate with predeposited platinum electrodes using a dispenser. The thickness of the sensing layer was of 10–12 μm. After drying at 90 °C for 1 hour, the substrate was heated to 750 °С for 15 minutes and kept thereafter at 400 °C for several hours. The measurements were carried out in a special chamber integrated together with a specially designed multichannel measuring device.

## Results and Discussion

### X-ray spectroscopy: Comparison of nanowire and nanopowder properties

**Figure 4:**
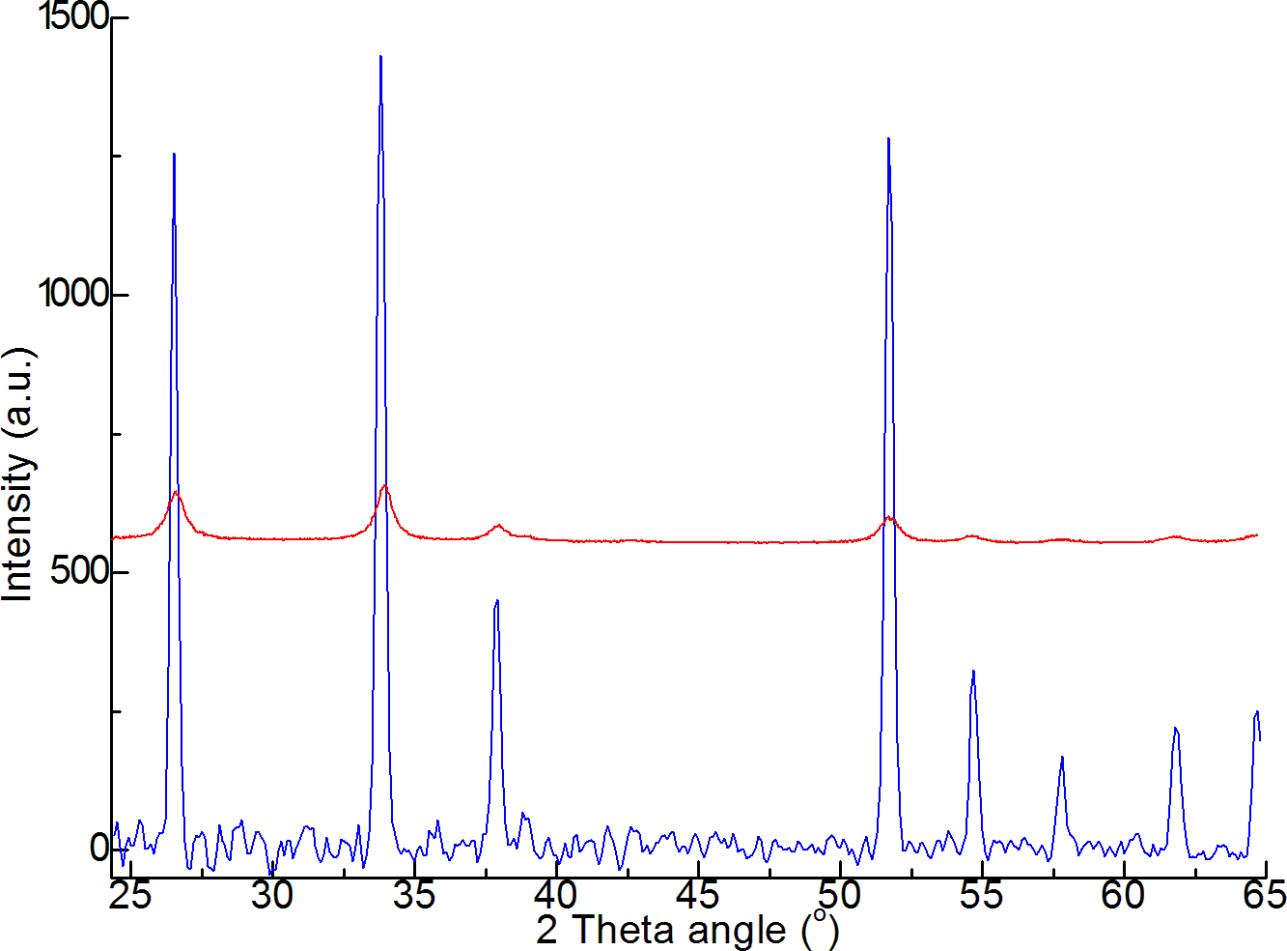
Experimental diffractograms of SnO2 nanowire (blue) and SnO2 nanopowder (red).

XRD spectra obtained by different methods (Figure 4) show a principal difference between the nanowires obtained by gas transport synthesis and the samples precipitated from nanopowder. The spectral band width at half-height characterizes the size of the coherence area, which is determined by the crystallinity of the structure. Nanowires formed by the vapor–liquid–solid mechanism have high and narrow peaks, confirming their monocrystalline structure. In contrast, the nanopowders exhibit low and wide peaks, demonstrating their disordered structure.

On the one hand, the disordered structure of the powder should lead to the appearance of large numbers of adsorption centers on the surface of the gas sensing material, thus increasing its sensing response. On the other hand, the monocrystallinity of the nanowires is a feature that increases the device stability.

### Synchrotron study of the nanowire and nanopowder samples

**Figure 5:**
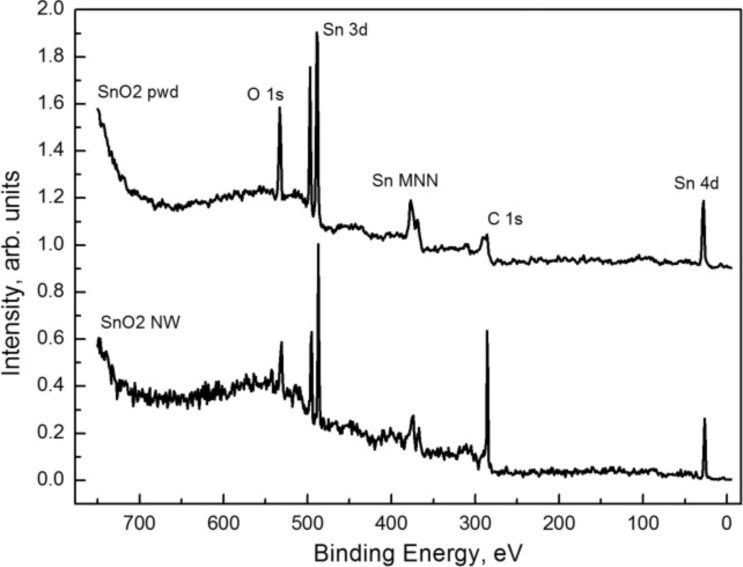
Photoelectron survey spectra for tin dioxide nanowires and powder samples obtained at an excitation energy of 800 eV.

The photoelectron survey spectra of SnO_2_ nanowires and SnO_2_ powder are given in Figure 5. All characteristic core levels of tin and oxygen atoms are marked together with the C 1s states of carbon groups. The purity of the samples is confirmed by the absence of impurity element lines. The position and single-component structure of the C 1s line for the nanowire sample is typical of the usual hydrocarbon contamination of ambient-stored samples. On powder samples, the same line exhibits a more complex fine structure as different forms of amorphous carbon are formed during material calcination.

**Figure 6:**
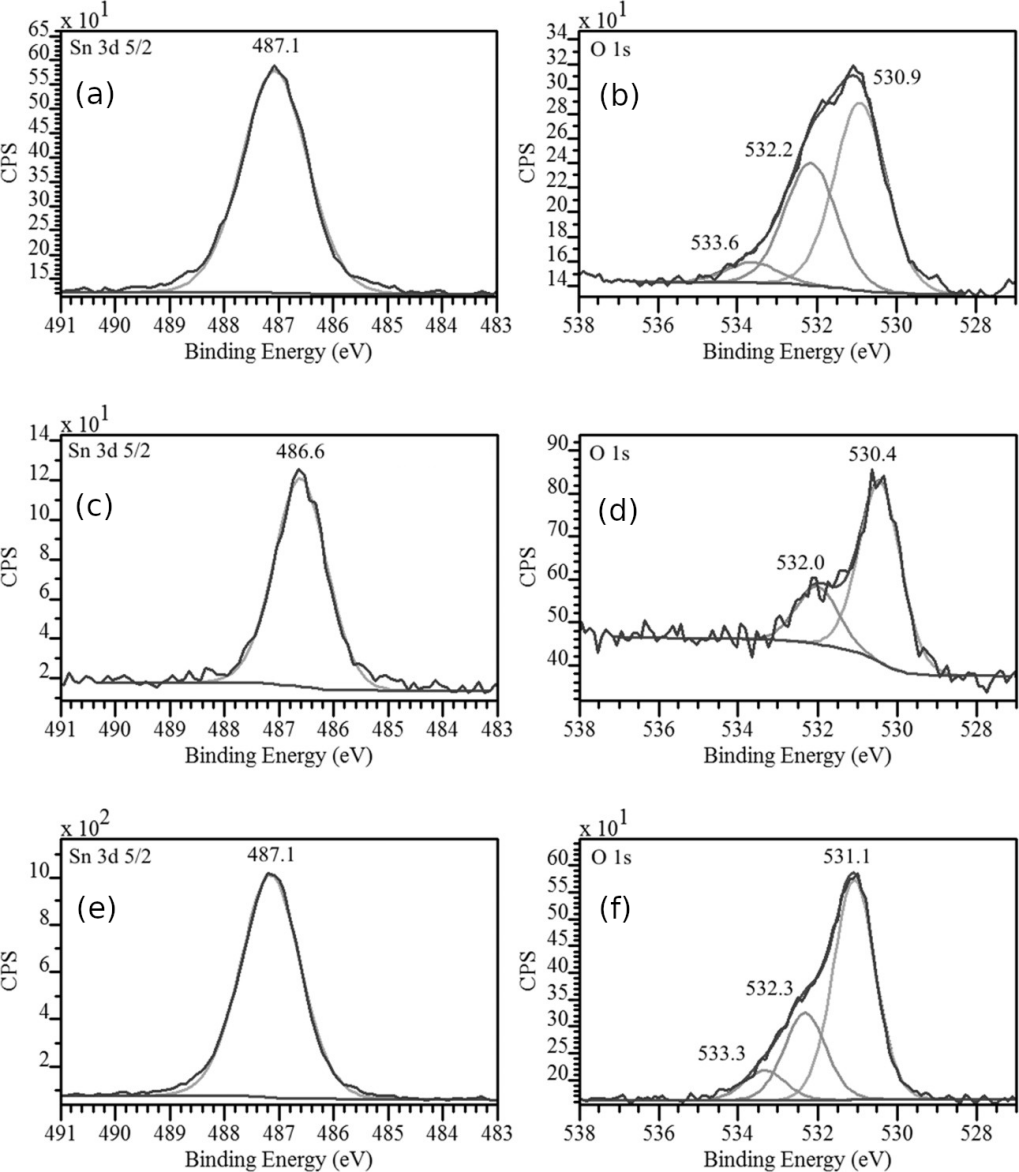
XPS spectra of (a) SnO_2_ powder, Sn 3d_5/2_; (b) SnO_2_ powder, O 1s; (c) SnO_2_ nanowires, Sn 3d_5/2_; (d) SnO_2_ nanowires, O 1s; (e) SnO_2_ sintered lump, Sn 3d_5/2_; (f) SnO_2_ sintered lump, O 1s.

Figure 6 represents core Sn 3d_5/2_ (left) and O 1s (right) lines of the samples. The binding energy for the 3d_5/2_ tin and oxygen 1s lines in the SnO_2_ reference occurred at 487.1 eV and 531.1 eV, respectively. In the powder sample (SnO_2_ pwd) the binding energy was in good agreement with the binding energy in the reference sample (487.1 and 530.9 eV, respectively). The core level binding energy in the wire sample was at lower values of 486.6 (Sn 3d_5/2_) and 530.4 (O 1s). These values were also observed on natural oxides formed on pure metallic tin surfaces (486.6 and 530.5 eV) [38,47]. These results are also in good agreement with previously published information about VLS grown tin dioxide nanowires with well-developed surfaces and physico-chemical states [38]. High energy components are observed for the 1s line of oxygen in the range of 532.0–533.6 eV (Figure 6). These components are usually caused by hydroxy groups and water molecules [38,47,50–51] adsorbed on the surfaces of the nanosized objects under study. The fine structure of the oxygen 1s level is considerably different for tin dioxide nanowires and for powder samples; this difference can be related to the contributions from the sorbed components. The O 1s component at 532 eV binding energy, prevailing on wire-like sample surfaces, was previously observed on metallic tin foil surfaces stored under laboratory conditions [38] and for magnetron sputtered tin nanolayers oxidized in air [47]. This component is also observed on the surfaces of nanopowder particles (see Figure 6) at about 532.2 eV and believed to be typical of sorbed OH^−^ ions [50]. These sorption processes are more noticeable in powder particles because the O 1s component, related to oxygen atoms bound with tin (530.4 eV), is a factor of two higher than for oxygen bound in OH^−^ ions (≈532 eV). The SnO_2_ nanowire sample, in comparison, showed the same component ratio as the SnO_2_ powder sample. Finally, the surface of the SnO_2_ powder sample contains water molecules; this follows from the low relative intensity of the 533.6 eV component (Figure 6) of the O 1s line [51]. Previously, this component was observed on polycrystalline nanolayers formed by magnetron sputtering of tin and ambient air oxidation afterwards [47].

**Figure 7:**
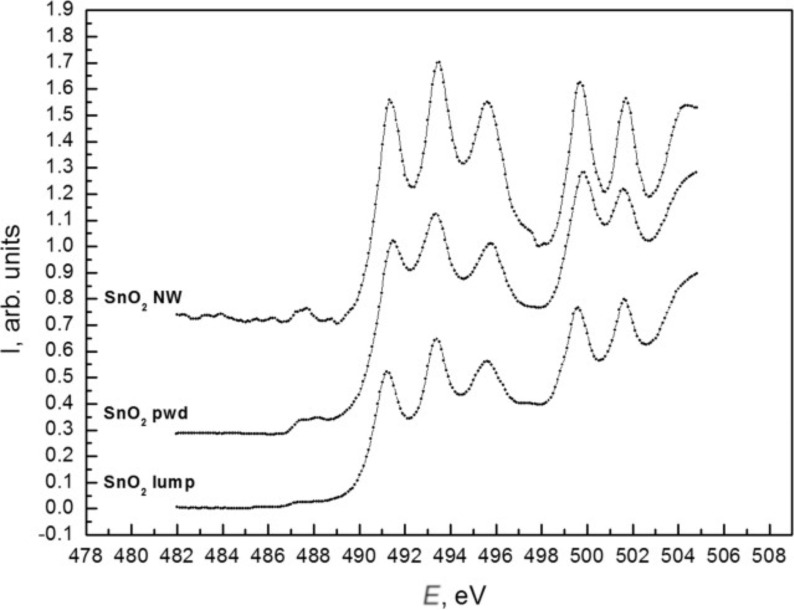
XANES Sn M_4,5_ spectra of SnO_2_ wire-like crystals (top) [37–38], SnO_2_ powder (middle) and sintered SnO_2_ lump reference samples (bottom).

Figure 7 compares XANES Sn M_4,5_ spectra of the samples with those obtained on the SnO_2_ sintered lump reference sample. From the analysis of the spectra fine structure, we can conclude that the wire-like sample is closer to the reference spectrum with a more pronounced Sn M_4,5_ absorption edge fine structure. Also, more noticeable is the "vacancy" feature at ≈487.5 eV observed in SnO_2_ nanowires, which is usually connected with the presence of oxygen vacancies [35,37–38]. The decrease in the half-widths and the increase in the relative intensity of the peaks for each of the features observed around 490 eV (main absorption edge of the SnO_2_) provides evidence for a more ordered structure in VLS grown single crystals of SnO_2_ nanowires than in calcinated and partially disoriented particles in powder samples.

In the SnO_2_ powder sample, a decrease is observed between the main Sn M_4,5_ XANES spectrum maxima and the more developed "vacancy" feature (≈487 eV). This smearing of the fine structure density of states is typical of native SnO_2-x_ oxide covering the surface of pure metallic tin foils [37–38] and was confirmed for SnO_2_ powder samples by the presence of noticeable amounts of oxygen vacancies (Figure 7).

**Figure 8:**
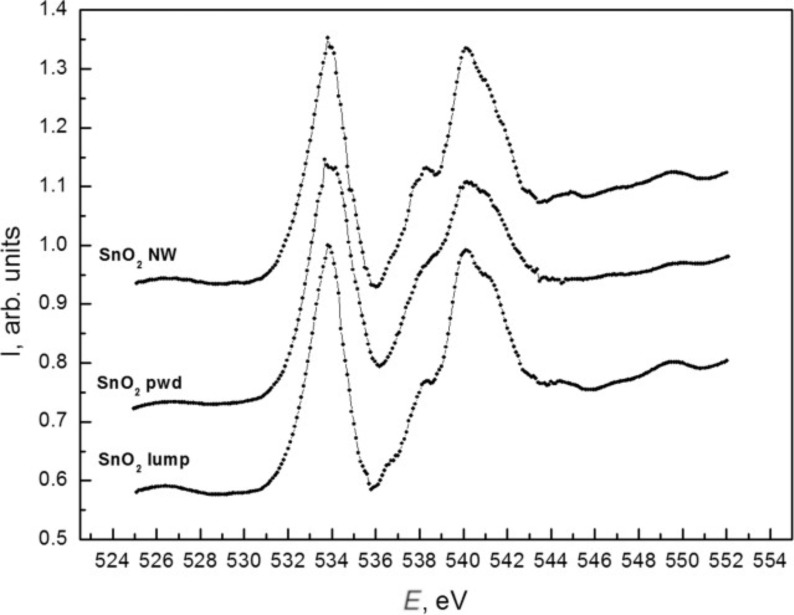
XANES O K spectra of SnO_2_ wire-like crystals (top) [37–38], SnO_2_ powder (middle) and sintered SnO_2_ lump reference sample (bottom).

XANES oxygen lines near K-edge spectra are presented in Figure 8. The fine structure distribution of the XANES O K spectra generally confirms the information obtained from tin absorption edge analysis. The relatively wider O K XANES spectra for the nanopowder in comparison to the SnO_2_ nanowire sample is caused by the better atomic ordering in wire-like single crystals and the less compact packing of SnO_2_ lattices in powder particles.

The XANES and XPS results do not contradict each other. The analysis depth of Sn M_4,5_ XANES is ≈10 nm, which is much larger than the Sn 3d_5/2_ XPS analysis depth (<2 nm at 800 eV synchrotron photon energy). It is clear that the inner parts of the SnO_2_ nanowires (Figure 7) consist of single crystals. These crystals have a very large surface, which can be similar to natural tin dioxide (see XPS data). At the same time, it is possible to assume the formation of core–shell structures in the case of SnO_2_ powder samples. Due to their special formation process, powder particles may contain noticeable amounts of oxygen vacancies in their volume. This assumption moves the electronic structure of SnO_2_ powder particles close to the bulk (core) of natural SnO_2-x_ oxides as confirmed by the XANES Sn M_4,5_ results (see Figure 7). After calcination, followed by continuous exposure to ambient lab conditions, the surface of SnO_2_ powder particles appears to be covered by a thin layer (shell) containing thermally cured vacancies. According to the observed XPS data (Figure 6), this shell is ≈2 nm of SnO_2_.

Thus wire-like crystals are covered by ≈1.5 nm of natural-like SnO_2-x_ while powder particles are covered by SnO_2_ nanolayers of the same thickness. The bulk of the wire-like crystals consists of crystalline SnO_2_. The powder particles consist of tin dioxide with the main structural unit packing character close to natural SnO_2-x_, which means that there is a noticeable density of oxygen vacancies inside the powder particles and at the surface of wire-like crystals.

### Gas sensing properties

During the characterization of the sensing properties of the devices, the following conditions were used: the sensors were exposed to air for one hour, then air was replaced by ambient conditions containing ammonia, where the sensor was also kept for one hour.

**Figure 9:**
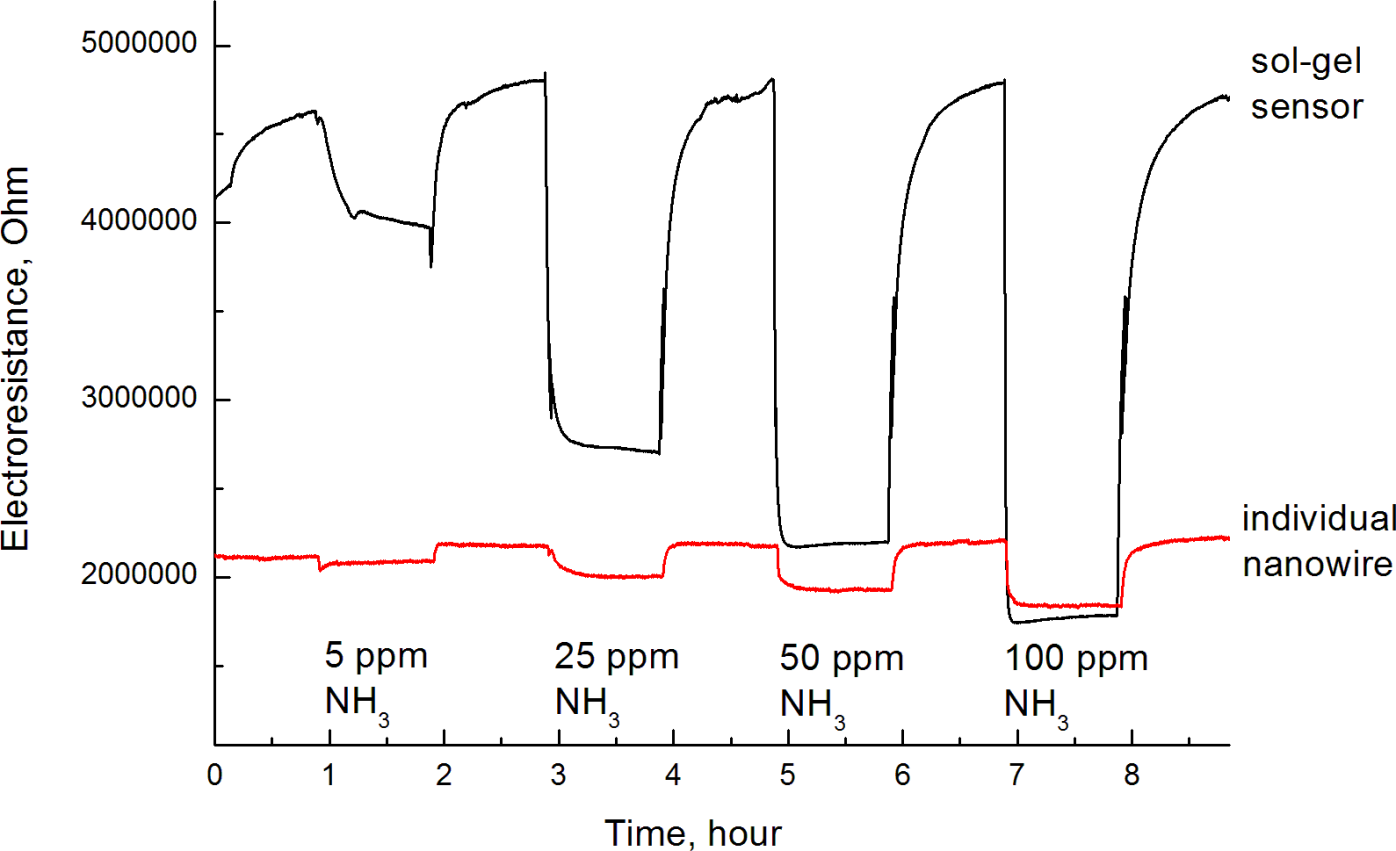
Response of nanowire and nanopowder sensors towards different concentrations of ammonia.

As shown in Figure 9, both sensing devices demonstrated stable readings, the background air resistance maintained a constant value, and long-term drift of the zero line was not observed. The response of the device manufactured by the sol–gel method is several times higher than that of the individual nanowire device.

**Figure 10:**
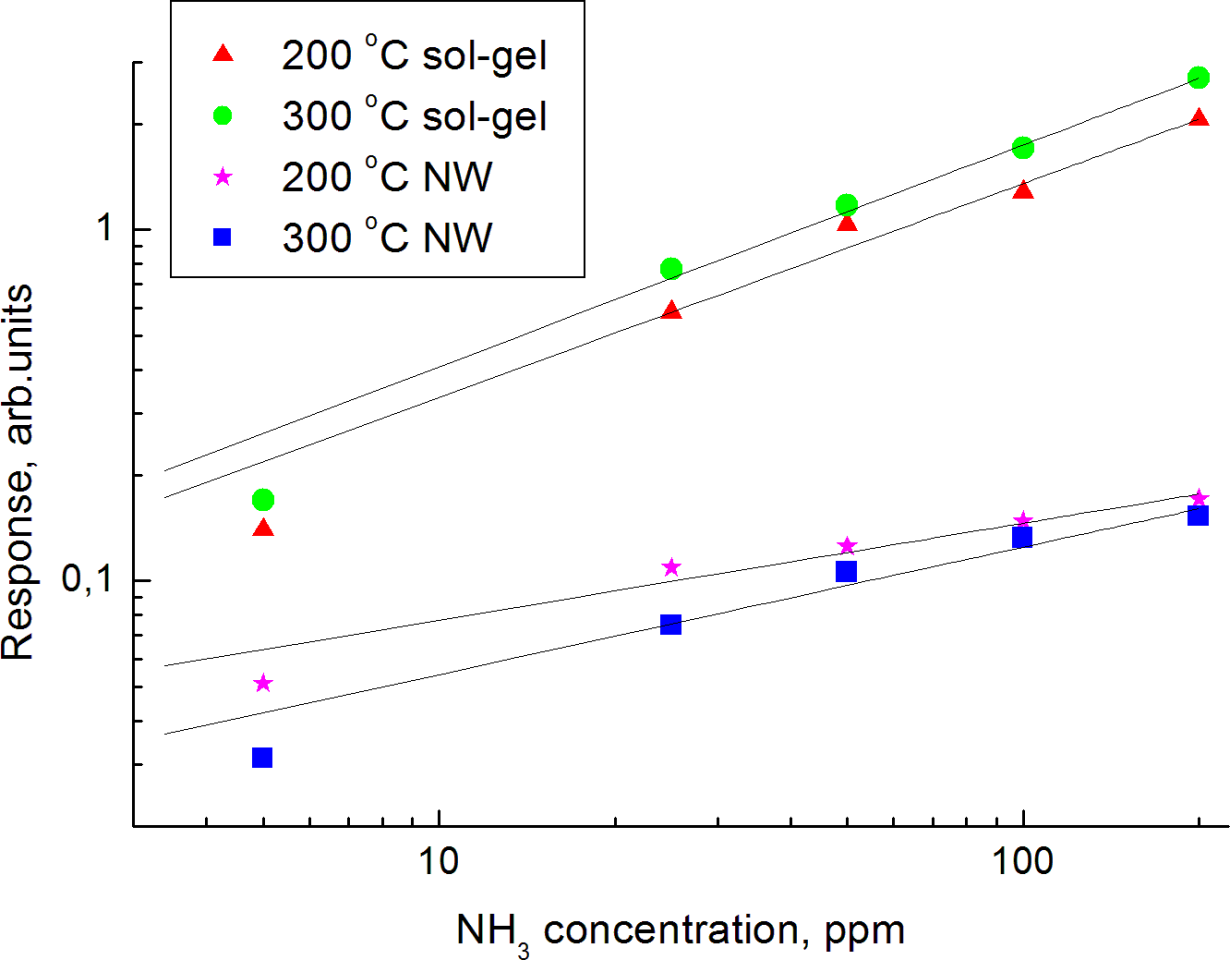
Calibration curves of the nanowire (NW) and sol–gel (nanopowder) sensors.

The sensor response, *S*, is defined as the relative difference of electrical resistance:

[6] $$S=\frac{R_{0}-R_{\text{x}}}{R_{\text{x}}},$$

where *R*_0_ is the sensor resistance in air, and *R*_x_ is the resistance when exposed to ambient in the explored medium. The sensor response as a function of ammonia concentration is shown in Figure 10.

These curves can be well described by power-law functions:

[7] $$S=a\cdot \varphi^{b},$$

where *a* and *b* are fit parameters and φ is the gas concentration expressed in ppm (Table 1).

**Table 1:** parameters of power function approximation of calibration curves.

Coefficients	Nanowire		Sol–gel sensor	
	200 °C	300 °C	200 °C	300 °C

*a*	0.03441	0.02348	0.08121	0.09541
*b*	0.30905	0.36284	0.61149	0.63107

As shown in Table 1, the sensors prepared by the sol–gel technique have higher values of the coefficient *a*, which is responsible for the resistance. In order to understand the reasons for this behavior, the specific adsorption at the nanowire surface was estimated. This estimation was carried out considering the geometry of the nanowire (diameter 70 nm, length 4 µm). The tin dioxide density is ≈7 g·cm^−3^, therefore the specific surface area is approximately 7 m^2^g^−1^. This is at least one order of magnitude smaller than the specific surface area of gas sensing materials obtained by the sol–gel method (≈120 m^2^g^−1^). The influence of the specific surface area on the sensitivity of the sensor is determined not only by the number of adsorption sites, but also by the electrical conduction mechanism. Smaller particles correspond to larger specific surface areas; the electrical conduction across such particles is more sensitive to an increase in the Debye layer width than by chemisorption.

The coefficient *b* of the power-law function also plays an important role, indicating the possibility of saturation at a high concentration of the test gas. In the sensors made by the sol–gel method, the coefficient *b* is much higher; such sensors therefore tend to saturate more easily. This phenomenon can be explained by two factors. The first one is the single crystal character of the nanowires. Uniform adsorption enthalpy of all the centers is typical of Langmuir-type adsorption, which is characterized by low power factors in the power-law approximation. Vice versa, the small size of crystallites obtained by sedimentation (Figure 2) should lead to a considerable dispersion of sorption enthalpies at the different sites. It is commonly thought that this fact results in an increase in the *b* factor. A second factor, which may lead to an increase in the *b* parameter value for sol–gel sensors is the multiplicity of current transfer routes. An increase (or decrease) of the Debye layer in individual nanowires may lead to a minimal (or maximal) current transfer. In sol–gel samples, multiple current transfer routes exist due to the percolation effect; therefore, it is almost impossible to achieve maximal or minimal values of current transfer.

**Figure 11:**
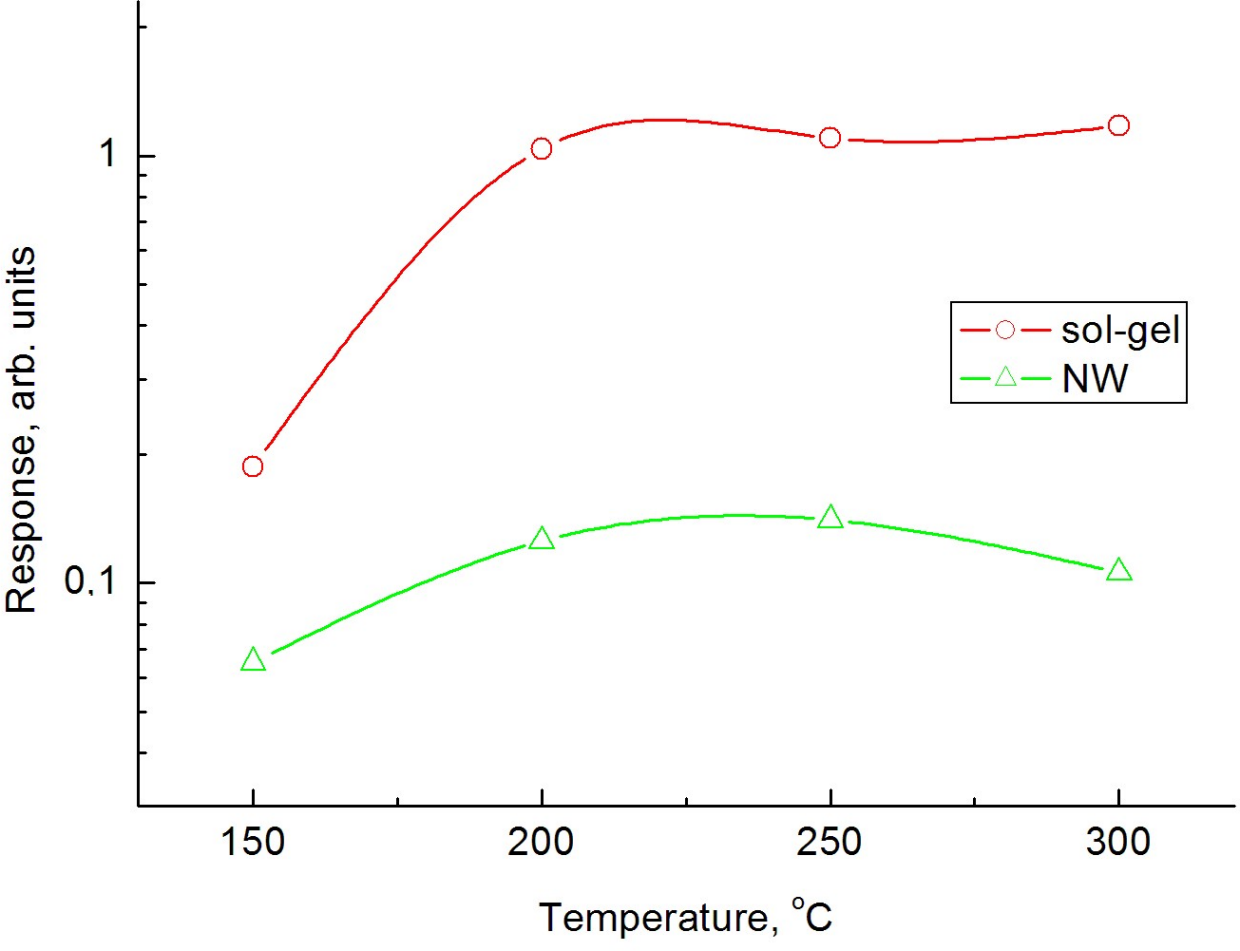
Response of two sensors based on sol–gel technology and on an individual nanowire (NW) as a function of sensor temperature.

As shown in Figure 11, a maximum response in both nanowire and sol–gel sensors is observed at a temperature of around 250 °C.

The effect of superficial hydroxy groups on the sensor cross-sensitivity to humidity was investigated in detail in [52]. It was shown that the application of tin dioxide materials synthesized by spark discharge and characterized as having a reduced concentration of superficial hydroxy groups resulted in a significantly decreased parasitic humidity response of the sensing material. The same effect is observed for the tin dioxide nanowires prepared by the dry gas-transport method.

## Conclusion

The presented results demonstrate that the sensors made by sol–gel technology are currently more sensitive in comparison with single-nanowire-based devices over a wide range of ammonia concentrations. Furthermore, they are simpler and cheaper to manufacture.

However, nanowire devices have also some key advantages. First, they are monocrystalline sensing materials, which provides for a greater stability in comparison with sol–gel sensors. It is known that the working surface of sol–gel sensors is continuously changing; some chemical bonds are being broken and others are being formed. This process leads to the continuous drift of sensor resistance, which distorts the signal. A monocrystalline surface is more stable, therefore resistance drift should be minimized in this case. A second advantage of individual nanowire sensors is the possibility of energy consumption reduction of the device when using a 4-electrode connection, where the outer pair of electrodes is used for applying the electrical potential. Another feature is the ability to use silicon technology for sensing device integration with measuring circuits. This allows the fabrication of an “e-nose” device in one chip.
